# Baitouweng Decoction Ameliorates Ulcerative Colitis in Mice Partially Attributed to Regulating Th17/Treg Balance and Restoring Intestinal Epithelial Barrier

**DOI:** 10.3389/fphar.2020.531117

**Published:** 2021-01-11

**Authors:** Zhiwei Miao, Liping Chen, Hui Feng, Mingjia Gu, Jing Yan, Yi Xu, Bai Ye

**Affiliations:** ^1^ Department of Gastroenterology, Zhangjiagang TCM Hospital Affiliated to Nanjing University of Chinese Medicine, Zhangjiagang, China; ^2^ Internal Medicine Department of Traditional Chinese Medicine, Zhongda Hospital Affiliated to Southeast University, Nanjing, China; ^3^ Department of Nephrology, Changshu Hospital Affiliated to Nanjing University of Chinese Medicine, Suzhou, China; ^4^ First Clinical Medical College, Nanjing University of Chinese Medicine, Nanjing, China; ^5^ Department of Gastroenterology, Affiliated Hospital of Nanjing University of Chinese Medicine, Nanjing, China

**Keywords:** ulcerative colitis, baitouweng decoction, Th17 cell, treg cell, intestinal epithelial barrier, inflammation

## Abstract

Ulcerative colitis (UC) is a chronic intestinal disease with unclear pathogenesis. With an increasing global prevalence over the past two decades, UC poses a serious threat to public health. Baitouweng decoction (BTW), a traditional Chinese medicine, has been shown to have good clinical efficacy for treating intestinal inflammation. Yet, the efficacy of BTW in UC and the underlying mechanism remain unclear. The current study aimed to determine whether BTW suppressed intestinal inflammation in mice and the potential mechanism. We used a dextran sulfate sodium (DSS)-induced murine colitis model to test the anti-inflammatory efficacy of BTW. Clinical symptoms were scored by the disease activity index (DAI), and the colon length and pathological changes in colon tissue were also used to further evaluate the efficacy of BTW. Precisely how BTW affected immune function and the intestinal barrier of UC mice was also examined. BTW significantly reduced DAI score and colonic pathological damage. BTW regulated the balance between T helper (Th)17 and regulatory T (Treg) cells, decreased interleukin (IL)-1β, IL-6, and tumor necrosis factor-α, and increased IL-10 levels. BTW reduced intestinal permeability of UC mice, increased expression of tight junction proteins (occludin and zonula occludens-1), and decreased expression of phospho-nuclear factor (p-NF)-κB and phospho-extracellular signal-regulated kinase (p-ERK) in the colon. BTW inhibited the ERK/p-NF-κB signaling pathway and suppressed expression of cyclo-oxygenase-2 and inducible NO synthase in lipopolysaccharide-activated RAW 264.7 cells. BTW significantly promoted the synthesis of short-chain fatty acids in the gut, particularly acetate, propionate, isobutyric acid, and isovalerate. The results suggest that BTW can protect against DSS-induced UC. The mechanism may be partially attributed to regulating the balance of Th17/Treg cells and restoring the intestinal epithelial barrier.

## Introduction

Ulcerative colitis (UC) is a state of chronic, recurrent intestinal inflammation presenting with abdominal pain, diarrhea, and mucopurulent stools as the key symptoms. Colonoscopy often reveals congestion, edema, granular changes, and ulceration of the intestinal mucosa. Histologically, UC is characterized by extensive ulcer distribution, with neutrophil infiltration and epithelial necrosis (Ungaro et al., 2017). The pathogenesis of UC is complex and unclear but is thought to be a combination of genetic susceptibility, external environmental stimulation, autoimmune dysfunction, and intestinal mucosal barrier damage (Conrad et al., 2014). In the 21^st^ century, the incidence of UC has increased globally, currently exceeding 0.3% in western countries by 2018 (Ng et al., 2017). UC has become a global public health threat and seriously affects patients’ quality of life (Sairenji et al., 2017). Currently, the main treatment strategies for UC include aminosalicylic acid (e.g., mesalazine and sulfasalazine), immunosuppressants (e.g., azathioprine), hormones (e.g., prednisone), and anti-tumor necrosis factor (TNF)-α antibody (e.g., infliximab), which have been shown to alleviate the symptoms UC but do not provide a definitive cure. However, long-term use of the aforementioned treatments is limited by adverse effects, including gastrointestinal reactions, bone marrow suppression, severe infection, and increased risk of lymphoma and cancer (Hauso et al., 2015; Ungaro et al., 2017; Bonovas et al., 2018).

Traditional Chinese medicine (TCM) is a reliable, alternative, and complementary therapy with fewer adverse effects. TCM has attracted worldwide attention for the treatment of many diseases (Lin et al., 2019). Baitouweng decoction (BTW), a well-known TCM prescription, has a documented history of >2000 years. Currently, it is widely used and is effective against various types of enteritis. BTW is composed of four TCMs: *Pulsatilla chinensis* (Bunge) Regel (Baitouweng), *Coptis chinensis Franch* (Huanglian), *Phellodendron chinense* C. K. Schneid (Huangbai), and *Fraxinus chinensis Roxb* (Qinpi). Previous studies have indicated that the extract of *Pulsatilla chinensis* (Bunge) Regel acts as an anti-inflammatory and immunomodulator in a mouse model of lipopolysaccharide (LPS)-induced systemic inflammation (Kang et al., 2019). Coptidine and berberine, which are the main components in *Coptis chinensis Franch*, have been shown to exert antitumor, anti-inflammatory, and antibacterial effects in rats (Wu J. et al., 2019; Zhu et al., 2019). An increasing body of evidence has highlighted the therapeutic effect of BTW, although little is known about the precise mechanism of action of BTW against UC. As a result, the current study investigated the mechanism by which BTW successfully suppressed gut inflammation in mice with dextran sulfate sodium (DSS)-induced UC.

## Materials and Methods

### Baitouweng Decoction Preparation

The raw herbs for BTW were purchased from Beijing Tongrentang Co. Ltd, with batch numbers: *Pulsatilla chinensis* (Bunge) Regel (20190815), *Coptis chinensis Franch* (20190723), *Phellodendron chinense* C. K. Schneid (20190820), and *Fraxinus chinensis Roxb* (20190901). They were identified by Dr. Yan Hui (School of Pharmacy, Nanjing University of Chinese Medicine, Nanjing, China.). Voucher specimens of the above four herbs (No. NJUCM_201909101-201909104) were stored in the Herbarium Center of Nanjing University of Chinese Medicine. *Pulsatilla chinensis* (Bunge) Regel 15 g, *Coptis chinensis Franch* 6 g, *Phellodendron chinense* C. K. Schneid 12 g, and *Fraxinus chinensis Roxb* 12 g were combined and submerged in distilled water (600 ml) for 30 min. Subsequently, the mixture was extracted twice (1 L first followed by 500 ml) over 1 h. Two rounds of extraction were conducted, and the resulting mixture was filtered. The filtrate was concentrated until the established drug content was 1 g/ml. To prepare the BTW extract used in the *in vitro* cell study, 10 ml BTW (1 g/ml) was lyophilized by a LAB-1A-50E freeze dryer (Biocool, Beijing, China), yielding 1.84 g powder that was used in the *in vitro* study.

### Liquid Chromatography Quadrupole Time-of-Flight Mass Spectrometry (LC-Q-TOF/MS)

Concentrated BTW (0.1 ml) was extracted by 1.0 ml methanol. The sample was then mixed and spun at 12,000 g for 5 min. The top layer was collected for LC-Q-TOF/MS analysis. The standard mixture, including esculin, fraxin, pulchinenoside A, coptisine, epiberberine, berberine, and palmatine (obtained from Chengdu Herbpurify Co. Ltd., Chengdu, China), was also prepared at the indicated concentration for LC-Q-TOF/MS analysis.

A Shimadzu UFLC 20ADXR system (Kyoto, Japan), with an online degasser quaternary pump, column temperature controller, and an autosampler, was used. The separation was achieved in a Thermo BDS Hypersil C18 (2.1 × 100 mm, 2.2 μm). The column temperature was set at 35 °C. The mobile phase was composed of 0.05% formic acid–water (phase A) and 0.05% formic acid–acetonitrile (phase B) with a flow rate set at 0.35 ml/min. The gradient elution program was as follows: 0–4.5 min, 10% B; 4.5–26 min, 10–30% B; 26–29 min, 30–80% B; 29–30 min, 80–10% B; 30–32 min, 10% B. The injection volume was 5 μL.

The raw MS and tandem MS (MS/MS) data were collected by a TripleTOF 5600 mass spectrometer. The analytes were ionized using electrospray ionization (ESI) in positive and negative modes. The m/z scan range for MS1 was 50–1200. For information-dependent acquisition, the m/z scan range for MS/MS was 50–1000. The main parameters for spectrometric analysis were as follows: ion source gases 1 and 2, 50 psi, DP, 55 eV, EP, 15 ev, and the collision energy, 15.

The concentrations of the main constituents (esculin, fraxin, epiberberinem, berberine, palmatine, coptisine, and pulchinenoside A) in BTW were also determined using a validated HPLC method (He et al., 2016) with a minor modification. Separation was achieved on an Agilent ZOBAX XDB-C18 column (250 × 4. 6 mm, 5.0 μm). A mobile phase that consisted of 0.1% phosphoric acid (A) and acetonitrile (B) and gradient elution were applied. The wavelengths were set at 310 nm for aesculin, 345 nm for fraxin, epiberberinem, berberine, palmatine, and coptisine, and 210 nm for pulchinenoside A. The chemical marker in *Pulsatilla chinensis* (Bunge) Regel (BTW) was pulchinenoside A. The chemical markers in *Coptis chinensis Franch* (Huanglian) and *Phellodendron chinense* C. K. Schneid (Huangbai) were shared and included epiberberinem, berberine, palmatine, and coptisine. The chemical markers in *Fraxinus chinensis Roxb* (Qinpi) were esculin and fraxin.

### Animals

Male C57BL/6 mice aged 6–8 weeks were obtained from the Qinglongshan Animal Breeding Farm (Nanjing, China) (certificate no. SCXK (Su) 2017-0001). Mice were housed in a sterile facility at 25 °C with a 12 h light/dark cycle and given sterile food and water. All studies were conducted under the authorization of the Ethics Committee of Animal Experiments.

### Induction of Colitis and Baitouweng Decoction Treatment

Sixty mice were randomized into five groups of 12 after 1 week of acclimation: 1) control group; 2) DSS group; 3) sulfasalazine (SASP)-treated group; 4) low-dose BTW-treated (L-BTW) group; and 5) high-dose BTW-treated (H-BTW) group. With the exception of the control group, all mice received 2.5% DSS (MP Biomedicals, Canada) in drinking water for 7 days. Subsequently, water was changed back to normal drinking water for an additional 3 days. The SASP group received 100 mg/kg SASP orally (Shanghai Sine Co. Ltd.) daily. The L-BTW and H-BTW groups were given 8 g/kg and 16 g/kg BTW orally, respectively. All doses were diluted with distilled water to ensure that each mouse received an equal volume of drug. The control and DSS groups were orally administered with an equal volume of distilled water.

To investigate whether the therapeutic effect of BTW on UC was related to the activation or inhibition of extracellular signal-regulated kinase (ERK), ERK agonist (TBHQ) and ERK inhibitor (U0126) were used alone or in combination with BTW to treat DSS-induced UC. Thirty-six mice were randomly assigned to six groups of six: 1) DSS group; 2) DSS + BTW group; 3) DSS + TBHQ group; 4) DSS + BTW + TBHQ group; 5) DSS + U0126 group; and 6) DSS + U0126 + BTW group. Induction of colitis was the same as above. BTW (16 g/kg) was given orally to the corresponding group. For TBHQ feeding, food pellets were mixed with 1% TBHQ (w/w; 97%, Sigma–Aldrich, St. Louis, MO, USA) (Jin et al., 2010). For U0126 treatment, 10 mg/kg U0126 (MedChemExpress, USA) was given intraperitoneally every 24 h (Bessard et al., 2008).

### Disease Activity Index

The overall performance of each mouse was assessed by specific parameters. Daily body weight was recorded along with any volume loss to bleeding or diarrhea. Body weight loss was recorded on a graded scale, where 0, 1 2, 3, and 4 represented no weight loss, 1–5% loss, 5–10% loss, 10–15% loss, and >15% loss, respectively. Stool consistency was recorded with 0, 1, 2, 3, and 4 representing normal, loose stools, mild diarrhea, diarrhea, and liquid stools, respectively. Fecal blood was quantitated on a scale from 0 to 4, where 0 represented no bleeding and 4 represented gross blood loss per rectum. The average of all scores was calculated and used to record DAI (Cooper et al., 1993).

### Colon Length and Spleen Coefficient

On day 10, all mice were killed by cervical dislocation. The colon from each mouse was collected and photographed, and the length was determined. A similar procedure was used to collect spleens, which were also weighed. The formula used to determine the spleen coefficient was spleen coefficient (%) = Ws/Wb × 100%, where Ws and Wb represent the spleen weight and body weight on day 10, respectively.

### Histological Analysis

Colon tissue was fixed in 4% (wt/vol) paraformaldehyde. Paraffin-embedded samples were processed to obtain 5 μm thick sections. Sections were stained with hematoxylin and eosin (H&E) and examined under light microscopy. The scoring criteria were used to determine the histological score (Van der Sluis et al., 2006). Data were collected and analyzed from eight independent samples.

### Cell Culture

RAW 264.7 macrophage cell line was purchased from the American Type Culture Collection (Manassas, VA, USA). The cells were grown in Dulbecco’s modified Eagle’s medium with 10% fetal bovine serum, 100 U/mL penicillin, and 100 μg/ml streptomycin and maintained at 37 °C in an environment containing 5% CO_2_. LPS (O111:B4; Sigma) was dissolved in sterile phosphate-buffered saline, and BTW and MEK-1/-2 inhibitor (U0126) were dissolved in dimethyl sulfoxide (DMSO). The final concentration of DMSO was 0.05% (v/v). RAW 264.7 cells were plated at 10^5^ cells/mL for 24 h and pretreated with BTW for 2 h prior to LPS (1 μg/ml).

### Cell Viability Assay

RAW 264.7 cells were plated at 10^4^ cells/well in 96-well plates per 100 μL medium. To determine the appropriate concentration that was not cytotoxic, the Cell Counting Kit-8 (CCK-8) was used (MedChemExpress, USA). CCK-8 solution (10 μL) was added to the cultured cells and the plates were incubated for 1–4 h. Metabolic activity was quantified by measuring light absorbance at 450 nm. Data were collected and analyzed from three independent samples.

### Nitrite Assay

Nitrite level in the medium was used as an indicator of NO production. RAW 264.7 cells were plated at 2 × 10^5^ cells/well in 96-well plates and incubated with or without LPS (1 μg/ml) in the absence or presence of BTW for 24 h. Each 50 μL of culture supernatant was mixed with an equal volume of Griess reagent (Beyotime, China) and incubated at room temperature for 15 min. NaNO_2_ was used to generate a standard curve, and nitrite production was determined by measuring optical density at 550 nm. Data were collected and analyzed from three independent samples.

### Cytokine Quantification by Enzyme-Linked Immunoassay

Whole blood was collected by orbital dislocation from all mice on day 10. The serum was centrifuged at 3500 rpm for 15 min to separate the serum. The serum concentration of interleukin (IL)-6, IL-10, TNF-α, and IL-1β was measured using commercial ELISA systems (BioLegend, San Diego, CA, USA). Data were collected and analyzed from six independent samples.

### RNA Isolation and Quantitative Real-Time Polymerase Chain Reaction

A total RNA Extraction Reagent (Vazyme Biotech Co. Ltd., China) was used to extract total RNA. HiScript II qRT SuperMix (Vazyme Biotech) was used to generate 1 µg of first-strand cDNA. PCRs were conducted with ChamQ SYBR qPCR Master Mix (Vazyme Biotech). The primers were purchased from Sangon Biotech (Shanghai) Co. Ltd., and the primer sequences (designed and checked by Primer Premier 6.0) were as follows: GAPDH-F, 5′-AGG​TCG​GTG​TGA​ACG​GAT​TTG-3′; GAPDH-R, 5′-TGT​AGA​CCA​TGT​AGT​TGA​GGT​CA-3′; TNF-α-F, 5′-CCT​CTC​TCT​AAT​CAG​CCC​TCT​G-3′; TNF-α-R, 5′-GAG​GAC​CTG​GGA​GTA​GAT​GAG-3′; IL-1β-F, 5′-CTC​GCC​AGT​GAA​ATG​ATG​GCT-3′; IL-1β-R, 5′-GTC​GGA​GAT​TCG​TAG​CTG​GAT-3′; IL-6-F, 5′-ACA​ACC​ACG​GCC​TTC​CCT​AC-′; IL-6-R, 5′-TCT​CAT​TTC​CAC​GAT​TTC​CCA​G-3′; IL-10-F, 5′-GCT​CTT​ACT​GAC​TGG​CAT​GAG-3′; IL-10-R, 5′-CGC​AGC​TCT​AGG​AGC​ATG​TG-3′; ZO-1-F, 5′-ACT​CCC​ACT​TCC​CCA​AAA​AC-3′; ZO-1-R, 5′-CCA​CAG​CTG​AAG​GAC​TCA​CA-3′; occludin-F, 5′-ACT​GGG​TCA​GGG​AAT​ATC​CA-3′; and occludin-R, 5′-TCA​GCA​GCA​GCC​ATG​TAC​TC-3′. Real-time PCR was conducted using a CFX Connect Real-Time PCR Detection System (Bio-Rad, Hercules, CA, USA). Parameters for amplification were as follows: 95 °C for 30 s, followed by 40 cycles at 95 °C for 10 s and 58 °C for 30 s. Relative mRNA expression was calculated using the 2^−ΔΔCt^ method (Livak and Schmittgen, 2001). Data were collected and analyzed from three independent samples.

### Protein Extraction and Western Blotting

The proteins from colonic tissues and RAW 264.7 cells were extracted using RIPA buffer (Beyotime) and protease inhibitor cocktail (200 mM AEBSF, 30 μM aprotinin, 13 mM bestatin, 1.4 mM E64, and 1 mM leupeptin in DMSO, 1:100) (Beyotime). Protein was then extracted following centrifugation. A BCA kit (Beyotime) was used to determine protein concentration. Subsequently, polyacrylamide gel electrophoresis (Bio-Rad) was conducted. The resulting membranes were replaced with polyvinylidene difluoride (Millipore, Billerica, MA, USA) before being submerged for 2 h in blocking solution. Primary antibodies against ZO-1 (1:1000), occludin (1:1000), cyclo-oxygenase (COX)-2 (1:1000), inducible NO synthase (iNOS) (1:1000), ERK 1/2 (1:1000), phospho (p)-ERK 1/2 (1:2000), nuclear factor (NF)-κB p65 (1:1000), p-NF-κB p65 (1:1000), β-actin (1:1000), and GAPDH (1:1000) (Cell Signaling Technology, China) solution were added to the membranes which were stored overnight at 4 °C. On the next day, the membranes were washed, and a secondary antibody was added for 1 h at room temperature. The secondary antibody was then washed away, and chemiluminescence was measured (ECL Plus; Beyotime). Data were collected and analyzed from three independent samples.

### Flow Cytometry Analysis

Mesenteric lymph nodes (MLNs) were lysed into single-cell solutions using collagenase type D (Roche, Basel, Switzerland) (1 mg/ml) and DNase I (Roche) (0.1 mg/ml) in Hanks’ Balanced Salt Solution for 30 min at 37 °C. The red cells in the preparation were lysed. After washing, the cells were processed for FCM (30). To identify T helper (Th)17 cells, plated cells were combined with 5 ng/ml phorbol myristate acetate along with 1 ng/ml ionomycin for 5 h. At 30 min, 10 ng/ml brefeldin A was added. Cells were washed and stained with 1 ml CD4-fluorescein isothiocyanate (FITC) per test, before being permeabilized with Cytofix or Cytoperm. Subsequently, 0.3 ml of IL-17A-phycoerythrin (PE) was added. For the detection of regulatory T (Treg) cells, a similar permeabilization protocol was used, but cells were stained with 0.3 ml forkhead box (Fox)p3-PE. Following washing, cells were then stained with CD4-FITC and CD25-APC, both at 1 ml per test. Finally, cells were rinsed using a buffer solution to remove any additional stain, and analysis was conducted in FCM buffer (Becton Dickinson, San Diego, CA, USA). Data were collected and analyzed from three independent samples.

### Determination of Short-Chain Fatty Acids in Feces

Concentrations of short-chain fatty acids (SCFAs) in mouse feces were determined using a previously reported method with minor modification (Huang et al., 2019). Briefly, 2 ml acetonitrile in a 1:1 volume ratio with water was combined with 40 mg of feces and mixed briefly for 5 min to extract SCFAs. The samples were spun at 12,000 g at 4 °C for 10 min. The supernatant (20 µL) was transferred into a separate tube, and 5 µg/ml internal standard (D3-hexanoic acid dissolved in a 1:1 ratio of water; Sigma–Aldrich) was added. The mixture was used for chemical derivatization. The samples were combined with 20 μL 200 mM 3-nitrophenylhydrazine in 50% acetonitrile and 20 μL 120 mM N-(3-dimethylaminopropyl)-N-ethylcarbodiimide HCl-6% pyridine in the same solvent. The mixture was incubated at 40 °C for 30 min. The solution was centrifuged at 12,000 g at 4 °C for 10 min, and 50 μL supernatant was collected and diluted by 50% aqueous acetonitrile to 100 μL for LC-MS/MS analysis.

A Shimadzu LC-20A system (Tokyo, Japan) was coupled with a 5500 QTRAP mass spectrometer (AB Sciex, Concord, ON, Canada). The LC system and MS system were connected with an ESI source. Chromatographic separation was achieved in a Thermo BDS Hypersil C18 column (2.1 × 100 mm, 2.4 μm). The column temperature was set at 40 °C, and the mobile phases consisted of 0.1% formic acid (A) and acetonitrile (B). A 19 min gradient elution program (with flow rate set at 0.35 ml/min) was used as follows: 0–2 min, 10% B; 2–9 min, 10–48% B; 9–14 min, 48–90% B; 14–16 min, 90% B; 16–17 min, 90–10% B; 17–19 min, 10% B. Samples were analyzed in negative ion mode. Multiple reaction monitoring mode was used, and the Q1/Q3 pairs were as follows: m/z 194.2 → 137.2 for acetate, m/z 208.1 → 165.1 for propionate, m/z 222.2 → 137.1 for isobutyrate and butyrate, m/z 236.1 → 137.1 for isovalerate and valerate, and m/z 253.2 →137.1 for internal standard (IS). The collision energy was set at −21, −20, −20, −24, and −26 for acetate, propionate, isobutyrate/butyrate, isovalerate/valerate, and IS, respectively. Data were collected and analyzed from eight independent samples.

### Immunohistochemical and Immunofluorescence Analysis

To quantitate the inflammation of colon tissues and function of the intestinal barrier, IF and immunohistochemistry (IHC) were used. Tissues were first dewaxed and rinsed. Subsequently, 0.01 M antigen was added along with sodium citrate buffer (pH 6). An IHC Biotin Block Kit was used to block endogenous biotin. Tissues were incubated with various primary antibodies at 4 °C overnight. Tissue slices were stained using a DAB kit along with hematoxylin after incubation using horseradish-peroxidase-conjugated antibodies for 1 h. For IF staining, Cy-3-labeled goat anti-rabbit IgG was used, and tissues were incubated for 1 h. Subsequently, 4′,6-diamidino-2-phenylindole (DAPI) was used to stain slices at room temperature for 10 min. Confocal microscopy was used to visualize tissues. Data were collected and analyzed from three independent samples.

### Intestinal Permeability

After mice were killed at the indicated time points, FITC–dextran (FD40) (Sigma) was used to assess intestinal permeability. FD40 was first dissolved in normal saline (50 mg/ml) and gavaged with 0.1 ml/10 g blood by body weight for 4 h. Separation of plasma was achieved using centrifugation. Plasma samples (40 μL) were combined with 160 μL normal saline. The concentration of FD40 was determined with fluorospectrometry at 485 nm (excitation wavelength) and 533 nm (emission wavelength). Data were collected and analyzed from four independent samples.

### Statistical Analysis

All results were presented in the form of mean ± SEM from two or three independent experiments as each experiment was conducted at least twice. One-way analysis of variance and Dunnett’s test were used to evaluate statistical significance between control groups and multiple dose groups. *p* < 0.05 was defined as statistically significant.

## Results

### Chemical Profile of Baitouweng Decoction

An in-house component library of BTW and MS1 and MS/MS data was compared to the literature or standards. More information was acquired under the anion mode when compared to the cation ion mode. The total ion chromatography of the standard mixture and BTW are shown in Supplementary Figure S1. A total of 32 compounds were identified (Table 1). The contents of aesculin, fraxin, epiberberinem, berberine, palmatine, coptisine, and pulchinenoside A in BTW (1 g/ml) were as follows: aesculin, 4.84 mg/ml; fraxin, 3.56 mg/ml; epiberberinem, 8.25 mg/ml; berberine, 18.37 mg/ml; palmatine, 7.49 mg/ml; coptisine, 2.19 mg/ml; and pulchinenoside A, 16.98 mg/ml.

**TABLE 1:** Main constituents identified in BTW.

No.	Mode	Retention time/min	MS^1^	MS^2^	Error (ppm)	Formula	Identification
1	N	3.16	339.0717	177.0179,133.0293	−1.3	C_15_H_16_O_9_	Esculin
2	N	3.79	353.08745	191.0555,161.0240,127.0401,	−1	C_16_H_18_O_9_	Chlorogenic acid
3	N	3.96	369.08212	207.0290,192.0058163.0040,135.0093	−1.6	C_16_H_18_O_10_	Fraxin
4	N	4.02	177.01951	149.0242,133.0289,107.0139	1	C_9_H_6_O_4_	Aesculetin
5	N	4.64	207.03004	192.0058,175.0039,147.0085,135.0089	0.7	C_10_H_8_O_5_	Fraxetin
6	N	5.01	367.10258	193.0503,191.0553,173.0454	−2.4	C_17_H_20_O_9_	5-O-Feruloylquinic acid
7	N	5.01	367.10258	193.0503,1910553,175.0397	−2.4	C_17_H_20_O_9_	4-O-feruloyl-D-quinic acid
8	P	5.28	320.09118	292.0930,262.0835,249.0756,2040790	−1.7	C_19_H_14_NO_4_	Coptisine
9	P	5.73	350.1387	334.1070,305.0673	−0.8	C_21_H_19_NO_4_	13-Methylepiberberine
10	P	6.74	336.12102	320.0873,292.0930,278.0775	−2.6	C_20_H_18_NO_4_	Berberine
11	P	6.68	352.1534	336.1191,322.1004,308.1249	−2.6	C_21_H_22_C_l_NO_4_	Palmatine hydrochloride
12	N	8.11	523.18127	403.1340,361.1287,223.0635,	−1.6	C_25_H_32_O_12_	Ligustroside
13	N	9.25	1119.5598	1073.5574,603.3896,469.1561	3.4	C_53_H_86_O_22_	3-O-α-L-arabinopyranosyl-23-hydroxybetulinic acid 28-O-α-L-rhamnopyranosyl(1→4)-β-D-glucopyranosyl
14	N	9.72	973.5029	927.4962,765.4447,603.3897	0.3	C_47_H_76_O_18_	Hederagenin 3-O-{β-D-glucopyranosyl(1→2) [β-D-glucopyranosyl(1→4)]-α-L-arabinopyranoside}
15	N	11.76	1249.6228	1203.6170,733.4535,469.1545	0.2	C_59_H_96_O_25_	3β-[(O-α-L-rhamnopyranosyl(1→2)-α-L-arabinopyranosyl) oxy]lup-20-(29)-en-28-oic acid28-O-α-L-rhamnopyranosyl(1→4)-O-β-D-glucopyranosyl(1→6)-β-D-glucopyranosylester
16	N	11.78	779.4584	587.3971	2.7	C_41_H_66_O_11_	Oleanolic acid 3-O-{O-α-L-rhamnopyranosyl-(1→2)-α-L-arabinopyranoside}
17	N	11.98	1235.6104	1189.6020,719.4334,469.1539	0.7	C_58_H_94_O_25_	3β-[(O-β-D-xylopyranosyl (1→2)-α-L-arabinopyranosyl) oxy]lup-20-(29)-en-28-oicacid 28-O-α-L-rhamnopyranosyl(1→4)-O-β-D-glucopyranosyl(1→6)-β-D-glucopyranosylester
18	N	12.02	841.4593	795.4554,471.3474	2.2	C_42_H_68_O_14_	3β,23-dihydroxy-lup-20 (29)-en-28-oicacid28O-β-D-glucopyranosyl(1→6)-β-D-glucopyranoside
19	N	12.29	1149.5744	1103.5658,633.3984,469.1560	1.3	C_54_H_88_O_23_	3-O-β-D-glucopyranosyl -23-hydroxybetulinic acid 28-O-α-L-rhamnopyranosyl(1→4)-β-D-glucopyranosyl(1→6)-β-D-glucopyranosyl ester
20	N	12.51	1103.5657	1057.5618,587.3965,469.1557	2.8	C_53_H_86_O_21_	Oleanolic acid 3-O-{O-β-D-glucopyranosyl-(1→4)-O-β-D-glucopyranosyl-(1→3)-O-α-L-rhamnopyranosyl-(1→2)-α-L-arabinopyranoside
21	N	12.82	811.4509	745.4378,603.3918	−2.9	C_41_H_66_O_13_	Hederagenin 3-O-{β-D-glucopyranosyl (1→4)-α-L-arabinopyranoside}
22	P	13.45	471.19898	453.1901,425.1950	−3	C_26_H_30_O_8_	Limonin
23	N	13.52	957.5101	749.4489	4.5	C_47_H_76_O_17_	Hederagenin 3-O-{O-α-L-rhamnopyranosyl-(1→2)-O-[β-D-glucopyranosyl-(1→4)]-α-Larabinopyranoside}
24	N	15.96	971.5244	925.517,469.1544,455.3513	0.4	C_48_H_78_O_17_	Betulinic acid 28-O-α-L-rhamnopyranosyl(1→4)-β-D-glucopyranosyl(1→6)-β-D-glucopyranosyl ester
25	N	16.41	781.4395	735.4307,603.3889,471.3451	−2.4	C_40_H_64_O_12_	3-O-β-D-xylopyranosyl (1→2)-α-L-arabinopyranosyl-23-hydroxybetulinic acid
26	N	16.64	749.44781	603.3853,471.3495,423.3253	−0.5	C_41_H_66_O_12_	Pulchinenoside A
27	P	16.92	455.20424	437.1930,411.2156,	−4.8	C_26_H_30_O_7_	Obacunone
28	N	17.51	941.51188	733.4552,455.3539161.0428	0.4	C_48_H_78_O_18_	Pulsatilloside C
29	N	17.54	895.5115	733.4551,587.3892,455.3585	4.4	C_47_H_76_O_16_	Oleanolic acid 3-O-{O-α-L-rhamnopyranosyl-(1→2)-O-[β-D-glucopyranosyl-(1→4)]-α-L-arabinopyranoside}
30	N	19.21	471.3466	407.3357	−2.9	C_30_H_48_O_4_	23-hydroxybetulinic acid
31	P	6.05	322.10648	307.0811,279.0859,249.0772	−2.8	C_19_H_16_NO_4_	Greenland coptisine
32	P	5.98	336.1211	320.0876,292.0933,262.0836	−5	C_20_H_17_NO_4_	Epiberberine

### Baitouweng Decoction Alleviated Colonic Injury in Dextran Sulfate Sodium-Induced Ulcerative Colitis in Mice

**FIGURE 1:**
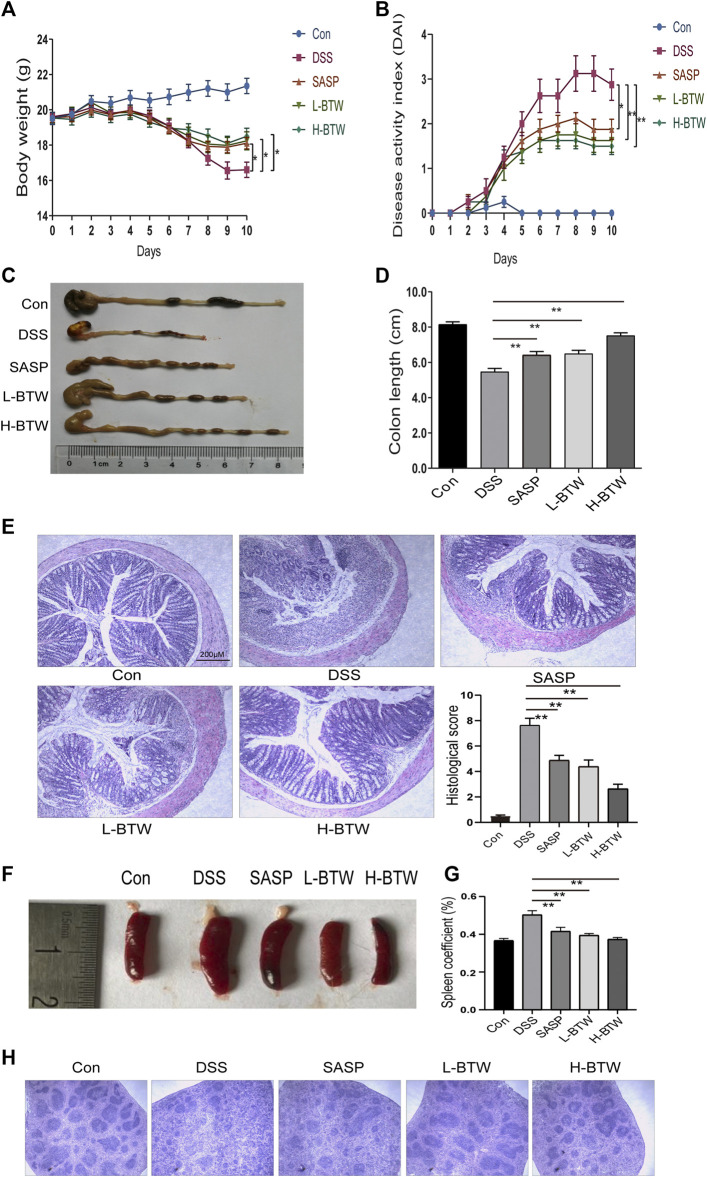
BTW alleviated DSS-induced UC. Body weight loss **(A)**, DAI score **(B)**, and shortened colon length **(C, D)** were reduced after L-BTW and H-BTW treatment. H&E staining and histological colitis score **(E)** showed that L-BTW and H-BTW reduced inflammatory cell infiltration in the colon. Representative pictures of spleen in each group **(F)**, spleen coefficient **(G)**, and H&E staining of spleens **(H)** showed that L-BTW and H-BTW alleviated splenomegaly caused by DSS, decreased the spleen coefficient, and reduced pathological damage of the spleen. Data are expressed as mean ± SEM (*n* = 8–12), *p < 0.05, **p < 0.01.

Acute colonic inflammation was induced by 2.5% DSS in drinking water for 7 days. The effect of BTW on UC was determined by assessing weight loss, colon length, histology of colon tissue, and DAI score. As expected, symptoms of diarrhea, mucus-like stools, fecal occult blood, bloody stools, weight loss, reduced activity, and poor hair color were associated with DSS treatment and subsequent colitis. DSS caused congestion, ulceration, edema, and shortening and crispness of colon in mice. However, treatment with SASP and BTW impeded disease progression, as shown by a lower degree of weight loss, decreased DAI, and improved colon length as compared with the DSS group (*p* < 0.01) (Figures 1A–D). Histological analysis showed a reduction in goblet cell density, mucosal thickening, destruction of architecture, infiltration of inflammatory cells, and epithelial cell damage in DSS-treated mice. All of the aforementioned parameters were reduced in a dose-dependent manner when treated with BTW. Furthermore, the histological score in L-BTW- and H-BTW-treated mice was significantly reduced when compared to that of the DSS group (*p* < 0.01) (Figure 1E).

### Effect of Baitouweng Decoction on Immune Organs in Ulcerative Colitis Mice

DSS-treated mice exhibited splenomegaly, which is commonly associated with immune dysfunction and increased inflammation (Liang et al., 2017). Spleen size was greater in the DSS group than in the control group. Treatment with BTW significantly reduced spleen volume (Figure 1F). The spleen coefficient was also reduced by BTW treatment (*p* < 0.01) (Figure 1G). DSS treatment decreased the red and white marrow of the spleen. This characteristic was reversed by SASP or BTW treatment (Figure 1H). These results suggest that BTW can reverse DSS-induced immune damage while protecting the architecture of the spleen.

### Effect of Baitouweng Decoction on Th17/Treg Cell Balance in Mesenteric Lymph Nodes

**FIGURE 2:**
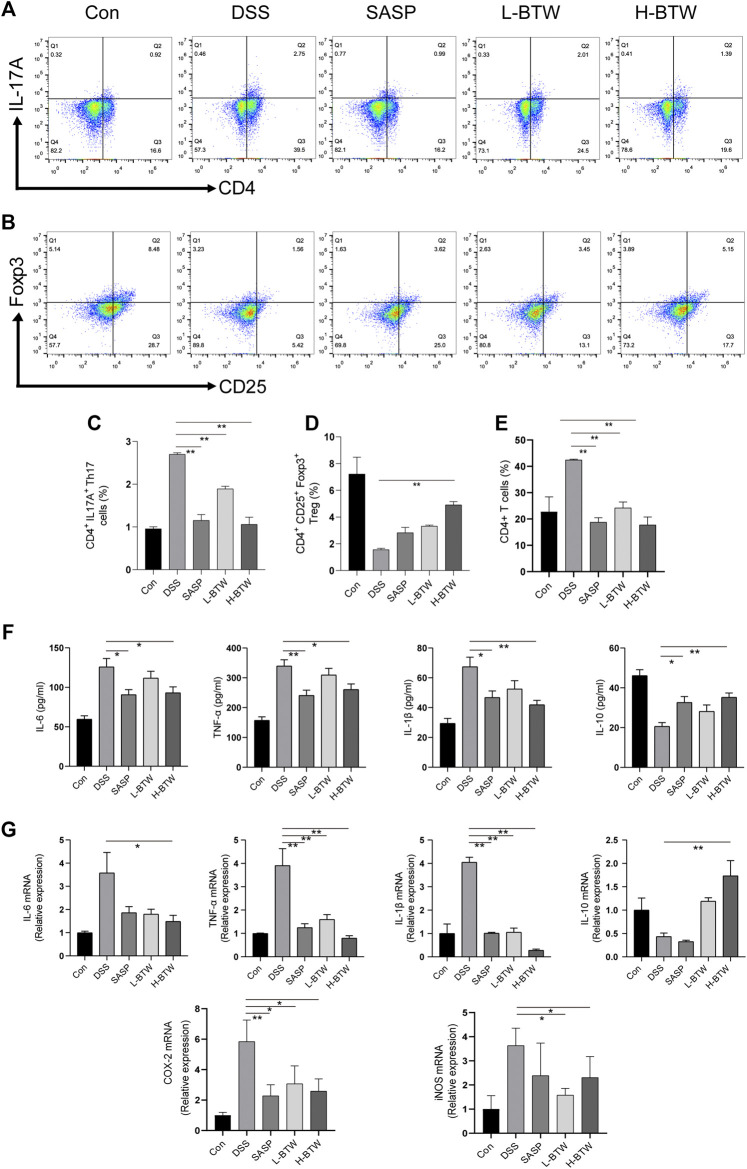
BTW regulated the balance of Th17/Treg cells and the level of pro-/anti-inflammatory cytokines in UC mice. Th17 and Treg cells in MLNs of all groups of mice were determined by FCM (*n* = 3). The gating strategy for FCM of Treg cells (CD25^+^Foxp3^+^), Th17 cells (CD4^+^IL-17A^+^), and CD4^+^ cells was set and populated using FlowJo software. L-BTW and H-BTW decreased the frequency of CD4^+^IL-17A^+^ cells in MLNs **(A, C)**, and H-BTW increased the proportion of CD4^+^CD25^+^Foxp3^+^ cells in MLNs **(B, D)**. L-BTW, H-BTW, and SASP significantly reduced the proportion of CD4^+^ T cells **(E)**. In addition, H-BTW downregulated IL-6, TNF-α, and IL-1β and upregulated IL-10 in serum of UC mice **(F)**, determined by ELISA (*n* = 6). H-BTW downregulated IL-6, TNF-α, IL-1β, COX-2, and iNOS mRNA and upregulated IL-10 mRNA in colonic tissue of UC mice **(G)** (*n* = 3), determined by RT-PCR. Data are presented as means ± SEM, *p < 0.05, **p < 0.01.

The literature shows a balance of Th17 and Treg cell functions in the development of colitis (Yang et al., 2017; Zhang et al., 2019). Therefore, the effect of BTW on Th17 and Treg cells was examined. The frequency of adaptive lymphocytic subset Th17 (CD4^+^ IL-17A^+^) in the MLNs of BTW- and SASP-treated mice was lower than that in the DSS-treated mice (*p* < 0.01) (Figures 2A,C). At the same time, the proportion of Treg cells (CD4^+^CD25^+^Foxp3^+^), which usually functions to suppress inflammation, was significantly higher compared with that in the DSS-treated mice (*p* < 0.01) (Figures 2B,D). CD4^+^ T cells were significantly increased by DSS but decreased significantly after BTW and SASP treatment (*p* < 0.01) (Figure 2E).

### Baitouweng Decoction Regulated Serum Proinflammatory and Anti-Inflammatory Cytokines in Ulcerative Colitis Mice

The imbalance of pro- and anti-inflammatory cytokine plays a key role in the development of inflammatory bowel disease (IBD) (Hur et al., 2012). Expression of proinflammatory cytokines such as TNF-α, IL-1β, and IL-6 associated with Th17 cell differentiation was significantly lower in the BTW group than that in the DSS group (*p* < 0.01 or *p* < 0.05). Expression of IL-10, an anti-inflammatory cytokine secreted by Treg cells, was significantly upregulated in the H-BTW group compared with the DSS group (*p* < 0.01) (Figure 2F). A similar trend was observed in the expression of mRNA in colon tissue (*p* < 0.01 or *p* < 0.05) (Figure 2G).

### Baitouweng Decoction Treatment Restored the Intestinal Epithelial Barrier in Ulcerative Colitis Mice

**FIGURE 3:**
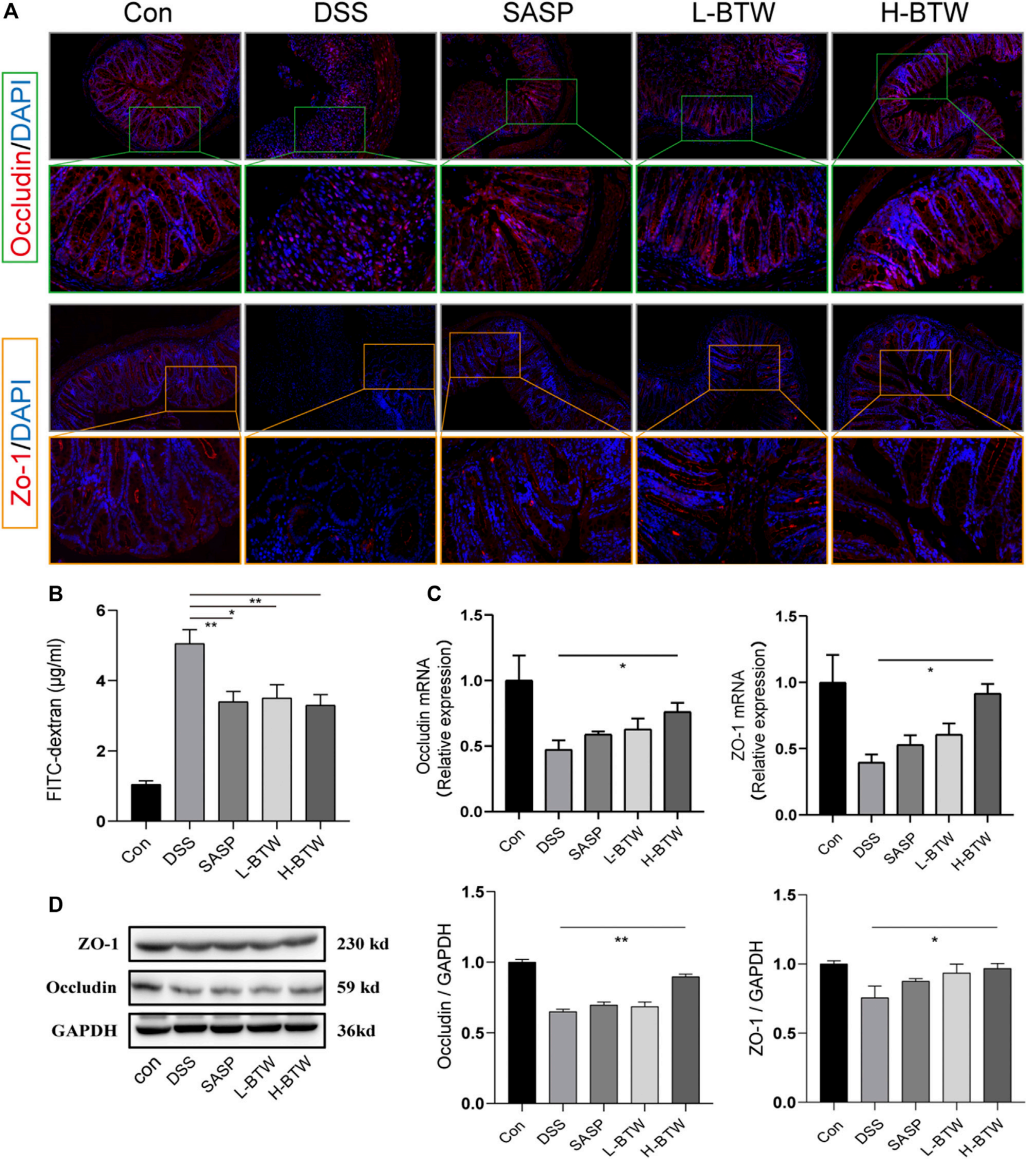
Effects of BTW on tight junction protein expression and intestinal permeability in UC mice. **(A)** Representative images of occludin immunostaining (red), ZO-1 immunostaining (red), and DAPI (blue) in colonic sections (×200 and ×500 magnification). **(B)** Intestinal permeability measurement by the FITC–dextran assay (*n* = 4). **(C)** Quantitative real-time PCR showed that occludin and ZO-1 mRNA were reduced in the DSS group but increased by H-BTW (*n* = 3). **(D)** Western blotting showed that protein expression of occludin and ZO-1 was reduced in the DSS group but significantly increased by H-BTW (*n* = 3). Data are expressed as means ± SEM, *p < 0.05; **p < 0.01.

To assess the potential protective effects of BTW on the function of the intestinal epithelial barrier, the intestinal permeability of FITC–dextran was determined. L-BTW- and H-BTW-treated mice showed lower intestinal permeability compared to mice given DSS without further treatment (3.5 ± 0.7, 3.3 ± 0.5 vs. 5.1 ± 0.7 μg/ml, *p* < 0.05, *p* < 0.01) (Figure 3B). We measured the tight junction proteins, an important structure for maintaining the mechanical barrier and permeability of mucosal epithelium. IF staining indicated that DSS reduced the expression of occludin and ZO-1 protein expression at the epithelial tight junctions (Figure 3A). BTW restored their expression levels. Western blotting indicated that H-BTW promoted the expression of occludin and ZO-1 compared to the DSS group without treatment (*p* < 0.01 or *p* < 0.05) (Figure 3D). To investigate this further, mRNA expression was determined using quantitative real-time PCR, which indicated that mRNA of occludin and ZO-1 was reduced by DSS but could be restored by BTW (*p* < 0.05) (Figure 3C).

### Baitouweng Decoction Suppressed Extracellular Signal-Regulated Kinase/NF-κB Activation in Ulcerative Colitis Mice

**FIGURE 4:**
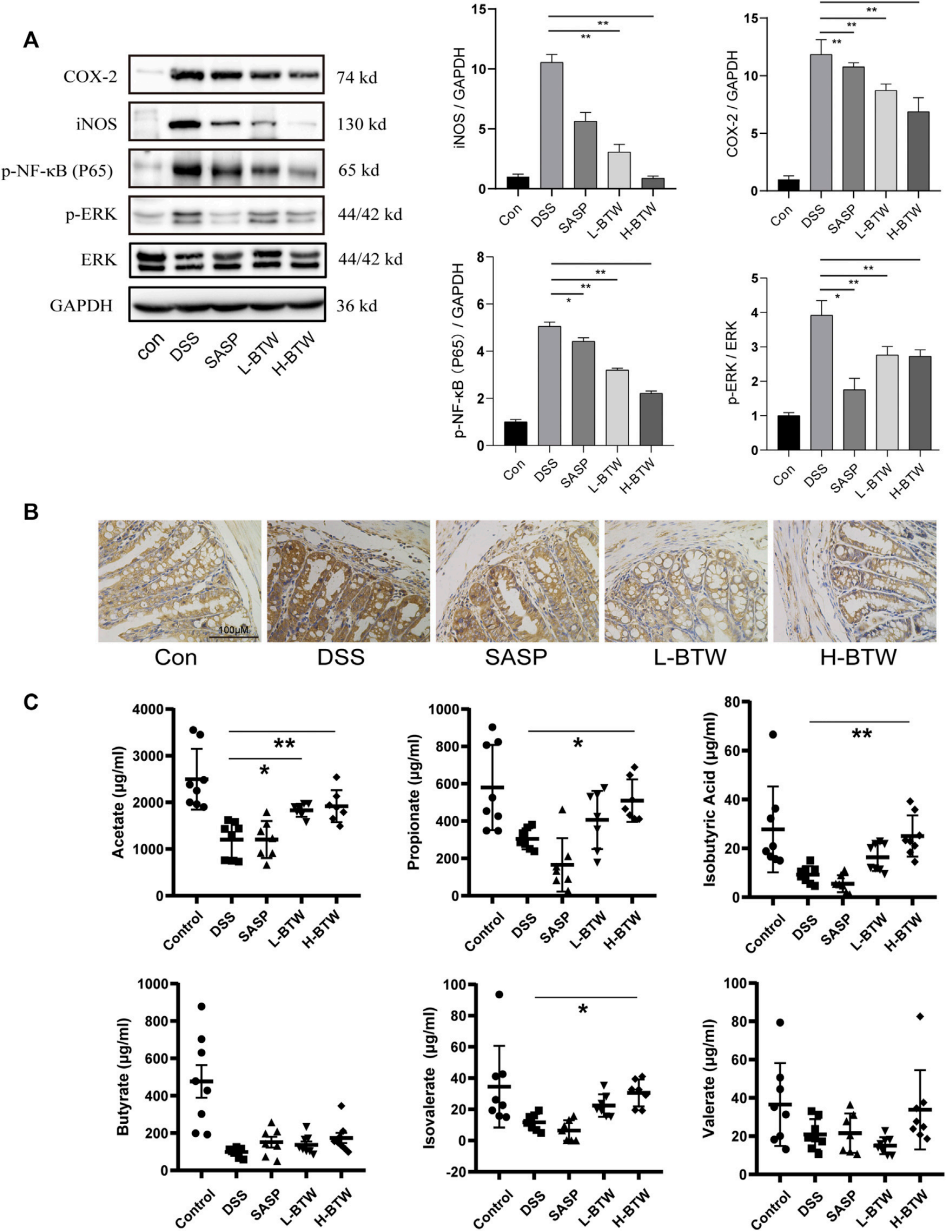
Effects of BTW on the activity of NF-κB/ERK in colon tissue and SCFAs in the gut of UC mice. **(A)** Phosphorylation of NF-κB and ERK was significantly increased by DSS but lowered by L-BTW and H-BTW; L-BTW and H-BTW significantly reduced protein level of COX-2 and iNOS, detected by western blotting (*n* = 3). **(B)** BTW decreased COX-2 expression in colonic tissue of UC mice, measured by IHC. **(C)** H-BTW significantly increased the concentration of acetate, propionate, isobutyric acid, and isovalerate compared with that in the DSS group (*n* = 8). Data are expressed as means ± SEM, *p < 0.05; **p < 0.01.

Previous studies have shown that NF-κB and ERK are related to downregulation of tight junction proteins (Sarkar et al., 2019; Yang et al., 2019; Jiang et al., 2020). Therefore, we determined whether BTW regulated phosphorylation of p65 and ERK. DSS increased the expression ratio of p-p65 and p-ERK. Remarkably, after BTW treatment, activation of p65 and ERK was significantly inhibited (*p* < 0.01) (Figure 4A). Simultaneously, the expression of proinflammatory mediators related to the NF-κB pathway, such as COX-2 and iNOS, increased significantly in the DSS group at mRNA and protein levels and was significantly decreased by BTW (*p* < 0.01 or *p* < 0.05) (Figures 2G and 4A).

### Baitouweng Decoction Increased Short-Chain Fatty Acids in Ulcerative Colitis Mice

Colonic bacteria play an important role in the processing of nondigestible dietary fibers that produce metabolites essential to the intestinal mucosa, such as SCFAs. SCFAs function as the main source of energy for colonocytes and in intestinal homeostasis by suppressing inflammation (Pascal et al., 2017; Parada Venegas et al., 2019). In patients with IBD, loss of microbiome diversity and the associated changes in SCFA levels are a primary source of pathology, and several treatment methods have been investigated to restore this balance (Pascal et al., 2017). In the current study, the composition of SCFAs was determined. The levels of key compounds such as acetate, propionate, butyrate, isovalerate, and isobutyric acid were all decreased in the DSS group compared to the control group (Figure 4C). However, BTW significantly increased the levels of acetate, propionate, isobutyric acid, and isovalerate compared to those in the DSS group (*p* < 0.01 or *p* < 0.05) (Figure 4C).

### Baitouweng Decoction Treatment for Ulcerative Colitis Depends on Regulation of Extracellular Signal-Regulated Kinase Pathway

**FIGURE 5:**
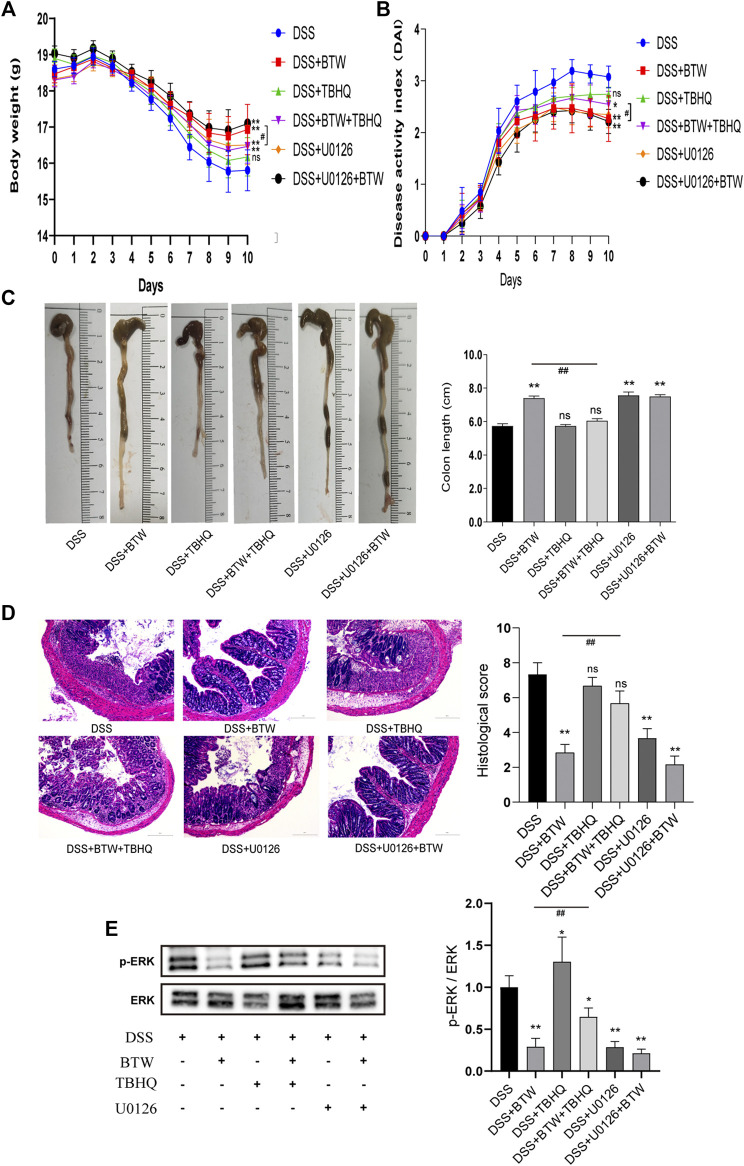
Effect of ERK regulators on UC mice. BTW and U0126 alone or in combination significantly improved body weight **(A)**, DAI **(B)**, colon length **(C)**, and colonic histological score of UC mice **(D)**, **p < 0.01 versus DSS group (*n* = 6). Combined use of TBHQ and BTW weakened the improvement of body weight **(A)**, DAI **(B)**, colon length **(C)**, and colonic histological score **(D)** of UC mice by BTW, #*p* < 0.05, ##*p* < 0.01 versus BTW group (*n* = 6). Western blotting **(E)** showed that treatment with BTW and U0126 alone or in combination significantly inhibited the activation of ERK, while TBHQ increased the activation of ERK compared with that in the DSS group, *p < 0.05; **p < 0.01 versus DSS group (*n* = 3). Combined use of TBHQ and BTW reduced the inhibition of ERK activity by BTW, ##*p* < 0.01 versus BTW group (*n* = 3). Data are expressed as means ± SEM.

ERK inhibitor U0126 downregulated ERK activity (Figure 5E) and showed an equivalent effect as BTW in improving body weight (Figure 5A), DAI (Figure 5B), colon length (Figure 5C), and colonic histological score (Figure 5D) of UC mice compared with those in the DSS group (*p* < 0.01). The combination of U0126 and BTW showed a tendency to improve the colonic histological score compared with BTW alone (*p* > 0.05). ERK agonist TBHQ increased the activation of ERK compared with that in the DSS group (*p* < 0.05); however, it did not aggravate the symptoms of UC, compared with those in the DSS group (*p* > 0.05). The combined use of TBHQ and BTW significantly reduced the inhibition of ERK activity (*p* < 0.01) and weakened the improvement of body weight (Figure 5A), DAI (Figure 5B), colon length (Figure 5C), and colonic histological score (Figure 5D), compared with BTW alone (*p* < 0.05 or *p* < 0.01).

### Baitouweng Decoction Inhibits Lipopolysaccharide-Induced NO Production in RAW 264.7 Cells

**FIGURE 6:**
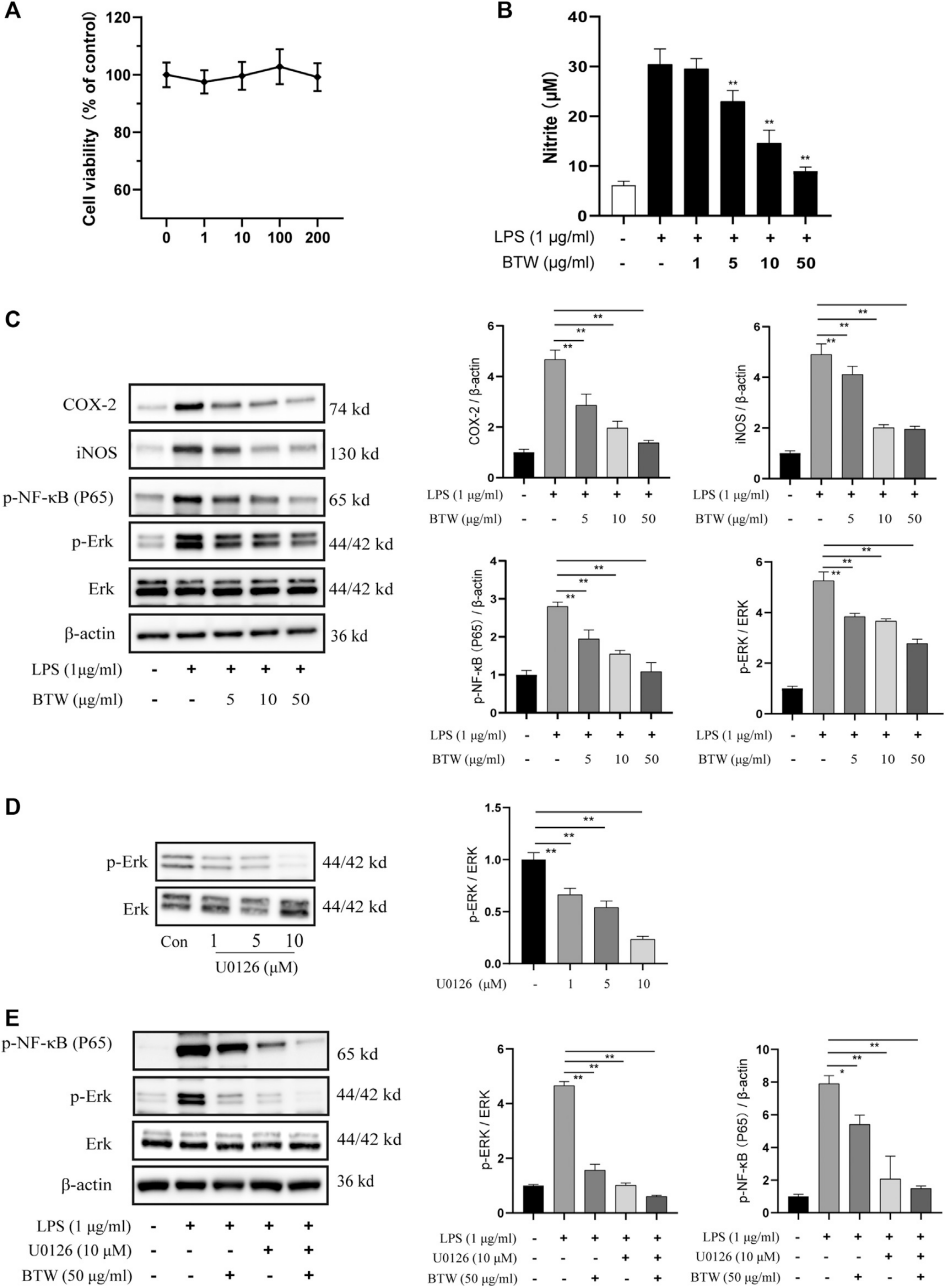
Effects of BTW on ERK/NF-κB pathway in RAW 264.7 macrophages. **(A)** Cells were treated with BTW (1, 10, 100, or 200 μg/ml) for 24 h. Cytotoxicity of BTW was measured using CCK-8 assay (*n* = 3). **(B)** Cells were incubated with or without LPS (1 μg/ml) in the absence or presence of BTW for 24 h. In the culture medium, NO production was measured by the Griess reaction (*n* = 3). **(C)** COX-2, iNOS, and phosphorylation of NF-κB and ERK were significantly increased by LPS but lowered by BTW (5, 10, or 50 μg/ml) in a concentration-dependent manner, detected by western blotting (*n* = 3). **(D)** Inhibition of ERK in RAW 264.7 cells after 24 h treatment with U0126 (1, 5, or 10 μM). **(E)** U0126 (10 μM) and BTW (50 μg/ml) significantly inhibited the activation of ERK/NF-κB induced by LPS (1 μg/ml), and this effect was enhanced by the combination of U0126 and BTW (*n* = 3). Data are expressed as means ± SEM, *p < 0.05; **p < 0.01.

Growth inhibition of RAW 264.7 by BTW was established by CCK-8 assay. BTW was not cytotoxic at any of the tested concentrations (*p* > 0.05) (Figure 6A). Based on the results, 1–50 μg/ml BTW was used for subsequent experiments. The effect of BTW on LPS-induced NO production in RAW 264.7 cells was investigated by measuring the release of nitrite in the culture medium by Griess reaction (Schmölz et al., 2017). The results indicated that BTW (5–50 μg/ml) inhibited LPS-induced NO production in a concentration-dependent manner (*p* < 0.01) (Figure 6B). We used up to 50 μg/ml BTW for further study.

### Baitouweng Decoction Inhibits the Activation of Extracellular Signal-Regulated Kinase/Nuclear Factor-κB in RAW 264.7 Cells Induced by Lipopolysaccharide

The effect of BTW on the activation of ERK/NF-κB in LPS-induced RAW 264.7 cells was determined by western blotting. LPS (1 μM) significantly increased the expression of p-ERK and p-p65. Pretreatment of cells with 5, 10, or 50 μg/ml BTW significantly decreased the activation of ERK and p65 in a concentration-dependent manner (*p* < 0.01) (Figure 6C). LPS increased the expression of COX-2 and iNOS, but this was decreased significantly in the BTW-pretreated (5, 10, or 50 μg/ml) group (*p* < 0.01) (Figure 6C).

Furthermore, we used U0126, an MEK1/2 inhibitor, to block the ERK-dependent pathway to verify the effect of BTW. As the concentration of U0126 increased, the inhibition of ERK increased gradually (Figure 6D). Pretreatment (1 h) with U0126 (10 μM) strongly inhibited the activation of ERK/NF-κB induced by LPS (*p* < 0.01), which was similar to the effect of BTW. The combination of U0126 with BTW augmented the inhibition of the ERK/NF-κB pathway (*p* < 0.01) (Figure 6E). This suggests that the ERK/NF-κB pathway is involved in LPS-induced inflammation in RAW 264.7 cells, and BTW acts as an inhibitor of the pathway.

## Discussion

BTW is a commonly used prescription and has been widely used as an adjuvant therapy for UC. Our previous meta-analysis based on clinical evidence showed that the use of BTW can significantly improve the clinical efficiency and endoscopic remission rate of UC (Miao et al., 2018). Although the use of BTW in UC has been recognized, the underlying mechanism remains unclear. The current study suggested that the regulation of Th17/Treg cell balance and repair of the intestinal mucosal barrier play a key role in the mechanism of BTW in protecting against UC.

To determine the effect of BTW on UC, parameters such as clinical symptoms and histopathology were used as indicators of drug efficacy (Peyrin-Biroulet et al., 2015). In our study, DSS was used to induce a model of colitis in mice, which is considered to be similar to the features of human UC (Bamba et al., 2012). We demonstrated that BTW showed a similar efficacy to SASP in improving symptoms such as body weight, DAI, and colonic shortening in mice. Moreover, the improvement induced by BTW showed a dose-dependent trend, especially in reducing pathological score.

To a certain degree, UC can be characterized as autoimmune dysfunction (Ungaro et al., 2017). Evidence suggests that UC is driven through a combination of immune hypersensitivity resulting in chronic inflammation and damage to the gut mucosa (Cader and Kaser, 2013). Previous studies have indicated that the balance between Th17 and Treg cells plays a key role in the pathogenesis of IBD and that restoration of this balance improves the symptoms of IBD (Maloy and Kullberg, 2008; Silva et al., 2016; Geng et al., 2017). In the current investigation, an increase in the Th17/Treg cell ratio in MLNs was found in mice treated with DSS. Treatment with BTW restored the ratio to a more normalized level. Expression of cytokines associated with Th17 cell differentiation, including IL-6, IL-1β, and TNF-α, was initially increased by DSS and subsequently improved with BTW treatment. Expression of IL-10, which is a key indicator of Treg cell differentiation, was inhibited in DSS-treated mice but upregulated by BTW treatment. We confirmed that the changes in the aforementioned cytokines were consistent with serum protein levels and colon mRNA levels. Moreover, we noticed that BTW inhibited the proliferation of CD4^+^ T cells in MLNs of UC mice. It is known that the activation of CD4^+^ T cells requires the involvement of transcription factor NF-κB, which activates immunity (Hsieh et al., 2002). BTW may affect CD4^+^ T cells directly or indirectly. Our results suggest that BTW may regulate autoimmunity and restore Th17/Treg cell balance, further affecting the expression of pro-/anti-inflammatory cytokines and thereby reducing intestinal inflammation in UC mice.

A large body of evidence suggests that intestinal mucosal barrier damage plays an important role in the development and maintenance of colitis (Hollander, 1999; Zeissig et al., 2007; Turner, 2009; Ahmad et al., 2017; Martini et al., 2017). Tight junction proteins, as a cytoskeleton, function to maintain the structure of epithelial cells and offer a line of antibacterial defense. Therefore, disruption of the tight junction could increase intestinal permeability and subsequently result in intestinal inflammation (Hollander, 1999; Turner, 2009). The normalization of intestinal permeability after drug treatment indicates disease remission (Arnott et al., 2002). In the present study, FD40 was administered orally and functioned as a permeability marker of the intestinal mucosal barrier (Volynets et al., 2016). BTW and SASP had similar effects on alleviating the changes in intestinal permeability caused by DSS. The levels of tight junction proteins (occludin and ZO-1) were significantly reduced by DSS but were markedly improved with BTW treatment. Previous studies have suggested that uncontrolled production of cytokines TNF-α and IL-1β can destroy the tight junction and increase permeability by activating myosin L chain kinase (Ye et al., 2006; Al-Sadi et al., 2010). BTW may protect the mucosal mechanical barrier indirectly by regulating immune-associated cytokines.

The literature suggests that the activation of NF-κB and ERK is associated with the activation of innate immunity and damage to the intestinal mucosal barrier (Al-Sadi et al., 2010; Yang et al., 2019). It has been widely shown that ERK1/2-dependent transcription factors, such as NF-κB, are key regulators in the pathogenesis of intestinal pathology through the production of inflammatory cytokines (TNF-α, IL-1β, and IL-6). Proinflammatory enzymes such as COX-2 and iNOS involved in the NF-κB pathway also play a key role in the pathogenesis of inflammation (Yang et al., 2019). It is reported that ERK differentially regulates Th17- and Treg-cell development and contributes to the pathogenesis of colitis, thus interfering with the ERK pathway, which could represent a therapeutic option for IBDs and other Th17-related autoimmune diseases (Liu et al., 2013). For example, Cortex Phellodendri Amurensis, derived from Phellodendron, reduces NO in LPS-stimulated RAW 264.7 cells through the inhibition of ERK 1/2, p38, and JNK phosphorylation, as well as inactivation of NF-κB (Choi et al., 2014). Our results are consistent with previous findings. We showed that *in vivo* phosphorylation of NF-κB p65 and ERK was significantly increased in the colon of mice with UC but was lowered by BTW. Application of ERK regulators showed that the therapeutic effect of BTW on UC was closely related to the inhibition of ERK activity. The combination of U0126 and BTW showed a tendency to improve the colonic histological score, compared with BTW alone. TBHQ weakened the inhibition of ERK activity by BTW, thus reducing the efficacy of BTW on UC. However, TBHQ as an activator of ERK did not aggravate DSS-induced colitis. It may be that TBHQ can also activate nuclear factor erythroid-2 related factor 2 (Nrf2)-mediated antioxidant response, which may partially neutralize the colonic damage caused by the activation of the ERK pathway (Xue et al., 2019). *In vitro*, BTW also inhibited the activity of NF-κB/ERK in LPS-activated RAW 264.7 cells. The combination of U0126 and BTW augmented the inhibition of activity of the ERK/NF-κB pathway. It is suggested that the effect of BTW on regulating immunity and protecting the intestinal mucosal barrier is related to the inhibition of NF-κB/ERK activity.

In recent years, it was believed that gut microbiome plays a key role in the development and progression of UC (Pittayanon et al., 2019). Furthermore, metabolites such as SCFAs have been proved to be involved in regulating immunity and maintaining the intestinal mucosal barrier (Parada Venegas et al., 2019). Herbal medicines are widely used to modulate the gut microbiome composition and regulate SCFA production (Feng et al., 2018). A recent study showed that Rhubarb Peony decoction regulated the gut microbiota and increased butyrate content, restoring the balance of Th17/Treg cells (Luo et al., 2019). Another study demonstrated that baicalin increased the abundance of SCFA-producing bacteria in the gut and protected intestinal integrity (Jia et al., 2019). In the present study, we also found that the fecal content of SCFAs (including acetate, propionate, isobutyric acid, butyrate, isovalerate, and valerate) in mice with UC was significantly reduced, which is consistent with previous studies (Luo et al., 2019; Wu D. et al., 2019). Strikingly, H-BTW treatment significantly improved the concentration of acetate, propionate, isobutyric acid, and isovalerate when compared with that in the DSS group. Some components of BTW, such as berberine and chlorogenic acid, have the potential to reverse intestinal flora imbalances (Nishitsuji et al., 2018; Jia et al., 2019). Therefore, the effect of BTW on SCFAs may be related to its regulatory effects in intestinal flora. This hypothesis was not examined in the current study, and further investigation in this direction could be beneficial.

The multiple therapeutic mechanisms of BTW could be attributed to its many active ingredients. LC-Q-TOF/MS analysis identified 32 compounds. Some of these monomers have been proven to have a suppressive effect in UC models. Aesculin is reported to act against UC by the regulating peroxisome proliferator-activated receptor-γ and NF-κB pathway (Tian et al., 2019). Chlorogenic acid has anti-inflammatory effects on DSS-induced colitis in mice by suppressing MAPK/ERK/JNK signaling and restoring intestinal flora ratio imbalance (Zhang et al., 2017; Gao et al., 2019). Fraxetin shows antioxidant properties and relieves intestinal inflammation (Witaicenis et al., 2014). Berberine has been used to treat UC by modulating gut microbiota and regulating Treg/Th17 cell ratio (Cui et al., 2018). Our *in vivo* and *in vitro* data can be confirmed by these previous findings. However, there are still many other ingredients that have not been reported, which will be the priority of our next study.

## Conclusion

BTW successfully inhibited colonic inflammation and suppressed the pathological changes in UC mice. The mechanism is partially attributed to regulating the balance of Th17/Treg cells and restoring intestinal epithelial barrier leakage. Based on these results, BTW is expected to be a promising treatment strategy for UC and other disorders.

## Data Availability Statement

The raw data supporting the conclusions of this article will be made available by the authors, without undue reservation, to any qualified researcher.

## Ethics Statement

The animal study was reviewed and approved by the Ethics Committee of Zhangjiagang TCM Hospital Affiliated to Nanjing University of Chinese Medicine.

## Author Contributions

ZM performed biochemical analysis and wrote the manuscript; HF and LC performed the animal experiments; MG and JY analyzed the data; BY and YX designed the research; ZM, YX, and BY prepared the manuscript and obtained funding for the project; and all authors read and approved the final manuscript.

## Funding

This work was supported by the Natural Science Foundation of Jiangsu Province, China (No. BK20191092); Jiangsu University Advantageous Discipline Construction Project (No. ZYX03KF058); Jiangsu Province Traditional Chinese Medicine Science and Technology Development Plan Project (No. RC201903); Suzhou Science and Technology Development Plan Project (No. SYSD2017007); and Zhangjiagang Science and Technology Project (No. ZKS1839).

## Conflict of Interest

The authors declare that the research was conducted in the absence of any commercial or financial relationships that could be construed as a potential conflict of interest.
