# Supplementary figures and images for: Baitouweng Decoction Ameliorates Ulcerative Colitis in Mice Partially Attributed to Regulating Th17/Treg Balance and Restoring Intestinal Epithelial Barrier
Source: Front Pharmacol. 2021 Jan 11;11:531117. doi: 10.3389/fphar.2020.531117 (PMC7883596; doi:10.3389/fphar.2020.531117)

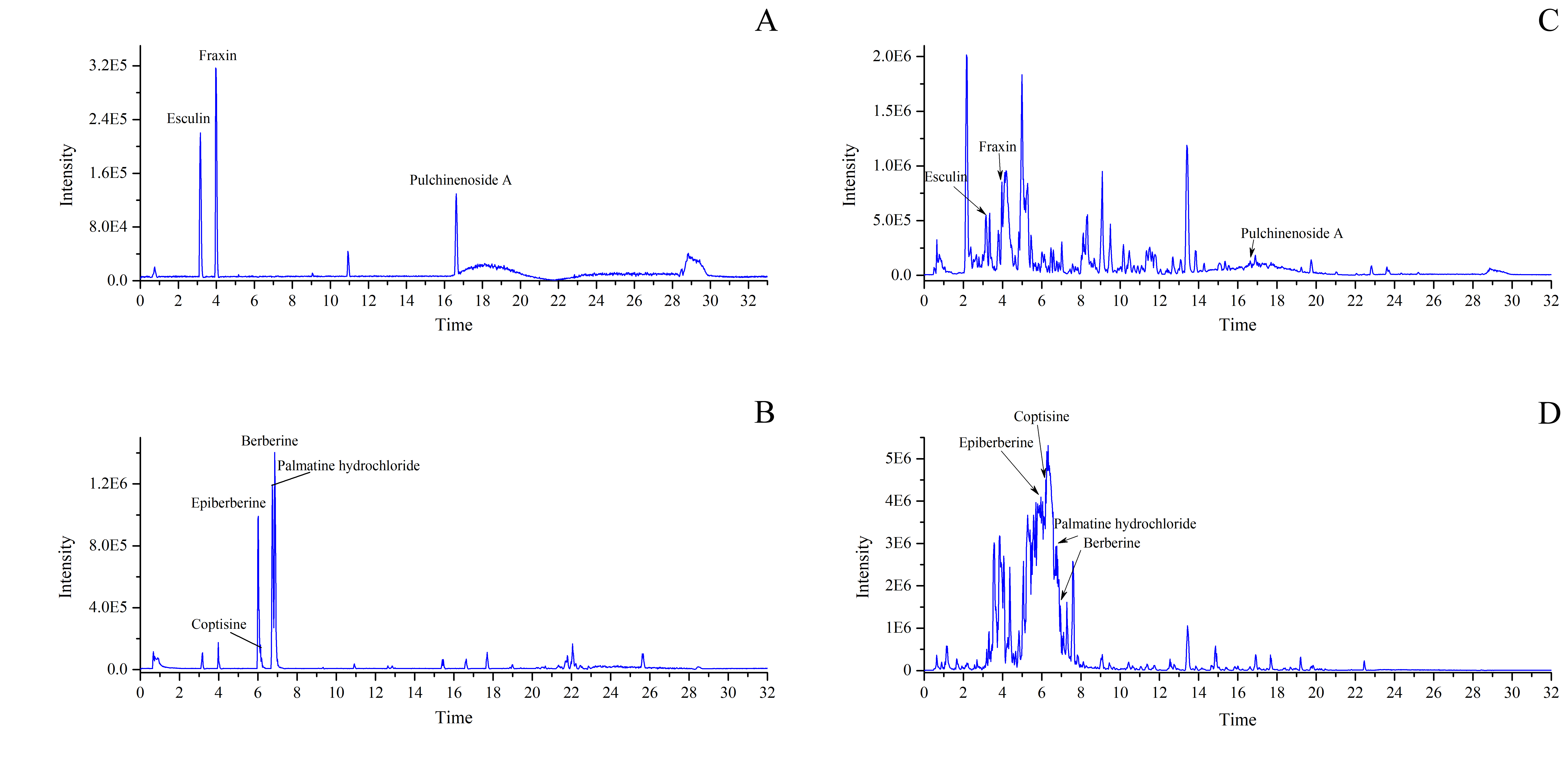

Supplement: Supplementary file 1 [file image1.tif]
